# The Efficacy of the Transversus Abdominis Plane Block in Abdominoplasty: A Systematic Review and Meta-Analysis

**DOI:** 10.7759/cureus.48992

**Published:** 2023-11-18

**Authors:** Nadia Taha, Lara Hodson, Kinseng Tong, Fadzlien Zahari, Zhi Liang Hoo, Yi Wah Wong, Shafiq Rahman

**Affiliations:** 1 Plastic Surgery, Leeds General Infirmary, Leeds, GBR; 2 Plastic Surgery, Queen Victoria Hospital, East Grinstead, GBR; 3 Urology, Harrogate District Hospital, Harrogate, GBR; 4 Elderly Medicine, St James University Hospital, Leeds, GBR; 5 Plastic Surgery, Northern General Hospital, Sheffield Teaching Hospitals NHS Foundation Trust, Sheffield, GBR

**Keywords:** transversus abdominis plane block (tap block), nerve block, reconstructive surgery, tap block, abdominoplasty

## Abstract

The transversus abdominis plane (TAP) block is a regional abdominal anaesthetic technique frequently used within non-cosmetic abdominal surgery. Its use in cosmetic abdominoplasty procedures is less frequently documented. The literature is devoid of a meta-analysis to quantitatively amalgamate the results of individual reports analysing the efficacy of TAP block compared to alternative analgesic methods in abdominoplasty surgery. The authors aimed to conduct the first meta-analysis within the literature to evaluate this technique’s effectiveness in abdominoplasties. Preferred Reporting Items for Systematic Reviews and Meta-Analyses (PRISMA) guidelines were referred to conduct this systematic review and meta-analysis. All observational and randomised controlled trials (RCTs) comparing the postoperative outcomes of patients who underwent abdominoplasties with TAP blocks versus no TAP blocks were included in this study. The time taken to first analgesia and the amount of analgesia used were the primary outcome measures. The secondary outcome measures include severity of pain, time to mobilisation, and length of stay. Random effect modelling was used for the analysis. The time taken to the first analgesic was significantly lower in the TAP cohort (p <0.05). In addition, there was a lower incidence of postoperative nausea/vomiting(OR 0.18, 95%CI 0.04 - 0.90, p=0.04). Mean total opioid use and operative time were comparable between the TAP and no TAP groups. A qualitative review of the visual analogue scale for pain amongst the included studies showed that it was lower in the TAP group. The authors report the first meta-analysis within the literature showing the efficacy of the TAP block in abdominoplasties. Further high-quality trials are recommended to further the current evidence base.

## Introduction and background

The transversus abdominis plane (TAP) block is a regional anaesthetic technique first introduced by Rafi in 2001 to alleviate postoperative pain in patients undergoing abdominal surgery [1]. This technique involves injecting a local anaesthetic agent into the neurovascular plane of the abdominal musculature, providing nerve blocks to the lower intercostal nerves, as well as the iliohypogastric and the ilioinguinal nerves between the subcostal margin and the iliac crest [1]. The TAP block is frequently used within general and obstetric-gynaecological surgery for common abdominal procedures, but its use in cosmetic abdominal surgery remains much less frequent [2,3].

Abdominoplasty is a common cosmetic procedure designed to excise excess skin and soft tissue from the lower abdomen to improve abdominal contour [4]. It can be performed in conjunction with liposuction to provide a more satisfactory aesthetic outcome for the patient [5]. Adequate postoperative analgesia allows for early mobilisation and adequate deep breathing, which have been shown to significantly reduce the rates of postoperative complications [6,7]. In addition to this, reducing postoperative opioid consumption has been shown to avoid the incidence of chronic pain, as well as mitigating the prevalence of long-term opioid misuse in postoperative patients [8-10]. A number of studies have reported on the efficacy of TAP blocks but, currently, the literature is devoid of a meta-analysis to quantitatively amalgamate the results of individual reports comparing the usage of TAP block to no usage post-operatively for abdominoplasty procedures [3,11-16]. The authors aimed to conduct the first meta-analysis within the literature to evaluate this technique’s effectiveness.

## Review

Methods

This systematic review and meta-analysis were performed as per the Preferred Reporting Items for Systematic Reviews and Meta-Analyses (PRISMA) guidelines [17].

Eligibility Criteria

All observational and randomised controlled trials (RCTs) of patients who underwent abdominoplasties comparing TAP versus no TAP blocks were included. Studies which involved ancillary procedures including liposuction were also included. There was no restriction on associated co-morbidities, age or sex. There was no exclusion on whether the TAP block was administered directly or under ultrasound guidance.

Primary and Secondary Outcomes

The primary outcome measures included the time taken to the first analgesic use as well as the number of tablets used. The secondary outcomes measured in this study will be the operating time, the incidence of postoperative nausea and/or vomiting, visual analogue scale (VAS) for pain, and the length of hospital stay in days.

Literature Search Strategy

Two authors (SR, FZ) independently searched the following electronic databases: MEDLINE (Medical Literature Analysis and Retrieval System Online), Embase, Google Scholar, CINAHL (Cumulative Index to Nursing and Allied Health Literature), and the CENTRAL (Cochrane Central Register of Controlled Trials). The last search was run on March 4, 2023. In addition, ClinicalTrials.gov (https://clinicaltrials.gov/ct2/home) was searched to screen articles. Our search strategy was restricted to studies with the English language. The search terminologies included “transverse abdominal plane”, “TAP”, “no TAP”, “subcutaneous infiltration”, “abdominoplasty” and “Fleur de Lys”. A review of the bibliographic lists of relevant articles was also conducted.

Selection of Studies

Two authors (SR, FSZ) assessed the abstract of articles identified from the literature searches independently. Articles that meet the eligibility criteria were selected following a robust screening of the full texts of the relevant reports. Discussion with a third author (YWW) was conducted in case of any discrepancies in study selection.

Data Extraction and Management

SR and FSZ created an electronic data extraction spreadsheet that is in line with Cochrane's data collection form for intervention reviews. A pilot-testing was done on the spreadsheet for articles that were selected randomly and adjusted accordingly. The data extraction spreadsheet included study-related data: first author(s), year of publication, study design, study size, the type of intervention, and comparison, baseline demographics of the included populations (age and gender), and primary/secondary outcome data of the studies. Two authors (NT, KST) collected and recorded the results. Consultation with a third author (LH) was done to resolve any disagreements in the process.

Data Synthesis

Review Manager (RevMan) Version 5.4 (Released 2020; The Cochrane Collaboration, London, United Kingdom) was used for data extraction and synthesis. The extracted data were entered into RevMan by two authors independently (NT, KST). The analysis involved was based on the random effect model. Forest plots with 95% confidence intervals (CIs) were used to report the results. The mean difference (MD) between the two groups was used to analyse the continuous outcomes.

Heterogeneity Assessment

The Cochran’s Q test (χ2) was used to assess heterogeneity among the studies. In addition, the calculation of I2 was done to quantify any inconsistency as an additional measure. Interpretation of this was guided as follows: 0-25% representing low heterogeneity, 25-75% representing moderate heterogeneity, and 75-100% representing high heterogeneity.

Risk of Bias Assessment

Risk of bias was assessed by one author (ZLH) using the Newcastle-Ottawa Quality Assessment Scale for observational studies, while the Cochrane Collaboration Tools for Risk of Bias was utilised for the RCTs [18].

Results

**Figure 1:**
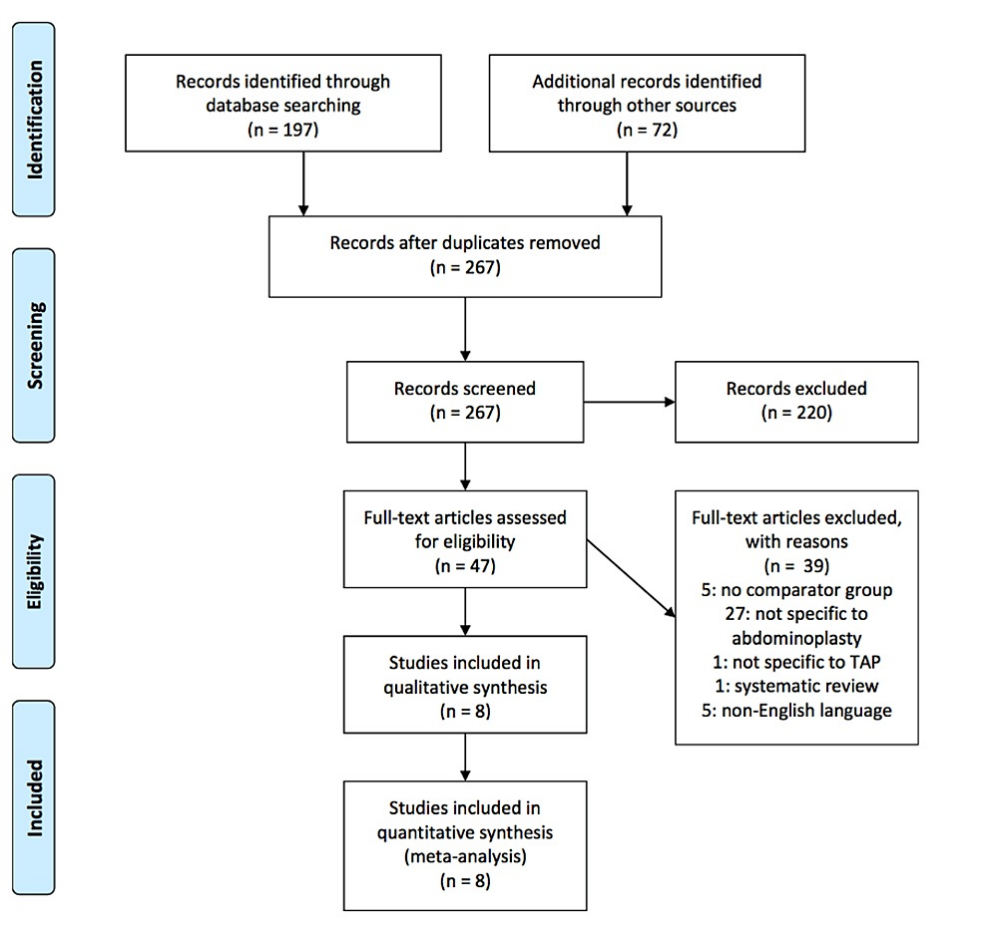
The 2009 PRISMA flow diagram for screening and selection of articles comparing efficacy of TAP block versus no TAP block in patients undergoing abdominoplasty. PRISMA: Preferred Reporting Items for Systematic Reviews and Meta-Analyses; TAP: transversus abdominis plane

The literature search process and results are demonstrated in Figure 1.

Eight studies were chosen based on eligibility criteria, and important information is summarised in Table 1.

**Table 1:** Summary of studies included in the data analysis for this study.

Author, year	Study design	TAP block (agent)	No TAP block (agent)	TAP block (n)/Mean age and sex distribution	No TAP block (n)/Mean age and sex distribution	Mean (SD) BMI with TAP block	Mean (SD) BMI without TAP block	Operative intervention
Araco et al., 2010 [13]	Case-control study	0.50% Bupivacaine	N/A	n=34 Mean (SD) age: 39 (8), F:34 M:0	n=41 Mean (SD) age: 42 (10), F:37 M:4	25 (2)	25 (2)	Abdominoplasty with flank liposuction
Sforza et al., 2011 [11]	Randomised controlled trial	0.50% Bupivacaine and 1% Lidocaine and adrenaline	Normal saline	n=14 Mean (SD) age: N/A, F:14 M:0	n=14 Mean (SD) age: N/A, F:14 M:0	N/A	N/A	Abdominoplasty
Fiala, 2014 [14]	Case-control study	0.25% Bupivacaine with Dexamethasone	0.25% Bupivacaine	n=16 Mean (range) age: 44.8 (25-67), F:15 M:1	n=16 Mean (range) age: 41.4 (26-65), F:15 M:1	N/A	N/A	Abdominoplasty with rectus plication
Abo-Zeid et al., 2018 [12]	Randomised controlled trial	0.25% Bupivacaine	0.25% Bupivacaine	n=16 Mean (SD) age: 40 (5.8), F:11 M:5	n=38 Mean (SD) age: 38 (4.9), F:9 M:7	30 (2)	29 (2)	Abdominoplasty
Salama, 2018 [16]	Randomised controlled trial	0.25% Levobupivacaine	Normal saline	n=30 Mean (SD) age: 41.6 (10.5), F:30 M:0	n=30 Mean (SD) age: 42.1 (9.8), F:30 M:0	28.2 (2.9)	26.5 (3.1)	Abdominoplasty
Gardner et al., 2019 [15]	Prospective, comparative study	0.25% Bupivacaine with dexamethasone	0.25% Bupivacaine	n=10 Mean (range) age: 43.8 (31-63), F:10 M:0	n=10 Mean (range) age: 38.8 (26-56), F:10 M:0	N/A	N/A	Abdominoplasty with core liposuction
Alotaibi et al., 2021 [3]	Randomised controlled trial	0.25% Bupivacaine	N/A	n=30 Mean (SD) age: 41.4 (7) F:30 M:0	n=30 Mean (SD) age: 42.6 (8), F:30 M:0	29.3 (2)	29.1 (1)	Lipoabdominoplasty with or without flank liposuction
Elsawy and Saeed, 2021 [19]	Randomised controlled trial	0.25% Bupivacaine	0.25% Bupivacaine	n=26 Mean (SD) age: 32 (4.1) F:8 M:18	n=25 Mean (SD) age: 31.88 (4.45), F:9 M:16	26.04 (1.59)	26..16 (1.55)	Abdominoplasty

Risk of Bias Assessments

The Newcastle-Ottowa Scale was used to evaluate the methodological quality of three observational studies. The results are shown in Table 2.

**Table 2:** Newcastle-Ottawa Scale quality assessment for the chosen articles.

Author, year	Selection	Comparability	Outcome	Total
Araco et al., 2010 [13]	☆☆☆☆	-	☆☆☆	7
Fiala, 2014 [14]	☆☆☆☆	-	☆☆☆	7
Gardner et al., 2019 [15]	☆☆☆☆	☆	☆☆	7

The Cochrane Collaboration tool for methodological quality review was used to assess the risk of bias in five RCTs (Table 3).

**Table 3:** Assessment of risk of bias in chosen studies using the Cochrane Collaboration tool. RCT: randomised control trials

Author, year	Bias	Author's judgement	Support for judgment
Sforza et al., 2011 [11]	Random sequence generation	Low risk	Randomised but method not declared.
	Allocation concealment	Some concerns	Blinding method not declared.
	Blinding of participants and personnel	High risk	Not feasible due to consent and operative nature.
	Blinding of outcome assessment	Some concerns	No mention of measures used to blind outcome assessors.
	Incomplete outcome data	Low risk	No incomplete data
	Selective reporting	Low risk	All outcome reported
Abo-Zeid et al., 2018 [12]	Random sequence generation	Low risk	Patient randomised but methods not declared
	Allocation concealment	Low risk	Closed envelope method used for randomization
	Blinding of participants and personnel	High risk	Not feasible due to consent and operative nature.
	Blinding of outcome assessment	Some concerns	No mention of measures used to blind outcome assessors.
	Incomplete outcome data	Low risk	No incomplete data
	Selective reporting	Low risk	All outcome reported
Salama, 2018 [16]	Random sequence generation	Low risk	Computer-generated numbers used for randomisation.
	Allocation concealment	Low risk	Sealed enveloped used.
	Blinding of participants and personnel	High risk	Not feasible due to consent and operative nature.
	Blinding of outcome assessment	Some concerns	Single-blinded RCT but no mention of measures used to blind outcome assessors.
	Incomplete outcome data	Low risk	No incomplete data
	Selective reporting	Low risk	All outcome reported
Alotaibi et al., 2021 [3]	Random sequence generation	Low risk	Computer-generated randomised chart used.
	Allocation concealment	Low risk	Computer-generated randomised chart used.
	Blinding of participants and personnel	High risk	Not feasible due to consent and operative nature.
	Blinding of outcome assessment	Some concerns	No mention of measures used to blind outcome assessors.
	Incomplete outcome data	Low risk	No incomplete data
	Selective reporting	Low risk	All outcome reported
Elsawy and Saeed, 2021 [19]	Random sequence generation	Low risk	Randomised but method not declared.
	Allocation concealment	Some concerns	Blinding method not declared.
	Blinding of participants and personnel	High risk	Not feasible due to consent and operative nature.
	Blinding of outcome assessment	Some concerns	Single blinded RCT but no mention of measures used to blind outcome assessors.
	Incomplete outcome data	Low risk	No incomplete data
	Selective reporting	Low risk	All outcome reported

Primary Outcome

**Figure 2:**
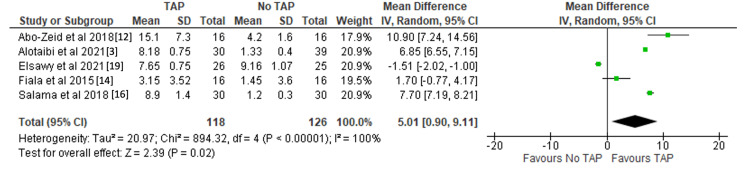
Forest plot demonstrating time to first analgesic (hours) with significance values included. References: [3,12,14,16,19] TAP: transversus abdominis plane

Time to first analgesic (hours): Five studies including 244 patients (118 TAP block; 126 no TAP block) reported the time to first analgesic requirement. Using a random effects model, the use of TAP block was associated with a significantly longer time before the first analgesic was required (MD 5.01 hours, 95%CI 0.90-9.11 hours, p = 0.02) [3,12,14,16,19]. Results are shown in Figure 2.

Secondary Outcomes

**Figure 3:**
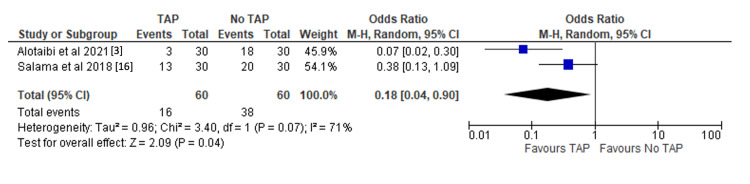
Forest plot demonstrating the incidence of postoperative nausea and vomiting with significance values included. References: [3,16] TAP: transversus abdominis plane

The incidence of postoperative nausea and vomiting: The use of TAP block is associated with a significantly lower incidence of postoperative nausea and vomiting (OR 0.18, 95%CI 0.04-0.90, p = 0.04). This is reported in two of the studies [3,16]. Results are shown in Figure 3.

**Figure 4:**
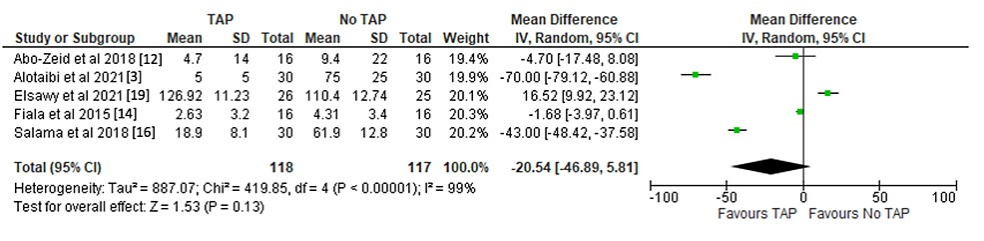
Forest plot demonstrating mean total opioid and narcotic usage (mg) with significance values included. References: [3,12,14,16,19] TAP: transversus abdominis plane

Mean total narcotic and opioid usage (mg): Five studies reported the mean total opioid use for analgesia. There was no significant difference between the use of TAP block and the absence of TAP block (MD -20.54 mg, 95%CI -46.89 mg to 5.81 mg, p = 0.13) [3,12,14,16,19]. Results are shown in Figure 4.

**Figure 5:**
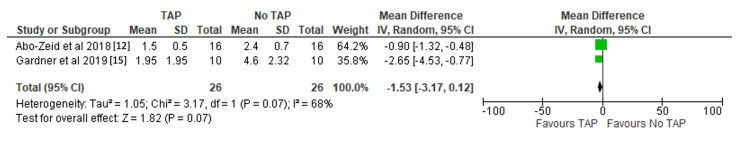
Forest plot demonstrating mean number of narcotic pills with significance values included. References: [12,15] TAP: transversus abdominis plane

Mean number of narcotic pills: The mean number of narcotic pills was comparable between both groups (MD -1.53, 95%CI -3.17 to 0.12, p = 0.07) [12,15]. Results are shown in Figure 5.

VAS: Pain score was reported according to a form of VAS in five studies, but different scoring systems were used [3,11,12,16,19]. Salama et al. reported the subjects’ satisfaction with their pain control using a seven-point Likert-like scale, ranging from 1 (strongly dissatisfied) to 7 (strongly satisfied) [16]. Pain scores at rest were similar whether a TAP block was used or not but were significantly lower during movement up to 24 hours postoperatively when a TAP block was used (p < 0.05) [16]. Sforza et al. recorded patients’ subjective pain scores using a scoring system of 0 (none) to 3 (severe) at 12 hours postoperatively [11]. Patients who received a TAP block reported a mean pain score of 1.2 as opposed to 1.5 in those who did not receive a TAP block (p < 0.001). Three papers used an 11-point scale ranging from 0 (no pain) to 10 (worst pain) [3,12,19]. Abo-Zeid et al. reported a significantly lower score at four hours postoperatively when a TAP block was performed, both at movement (4 vs 7.5; p < 0.05) and at rest (3 vs 5.5; p < 0.05) [12]. Alotaibi et al. demonstrated a mean VAS on the mobilisation of 1 +/- 1 (SD) in the TAP group as compared to 5 +/- 2 (SD) in the control group (p < 0.0001) [3]. Elsawy et al. found a significant difference in pain score at 12 hours postoperatively, with the TAP group at 3.92 +/- 0.64 (SD) and the control group at 4.50 +/- 0.51 (SD) (p = 0.002) [19].

**Figure 6:**
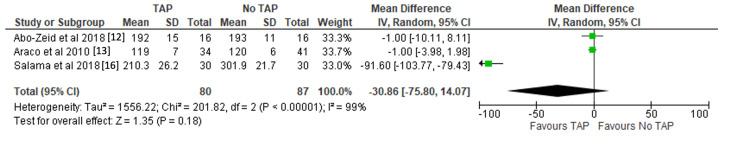
Forest plot demonstrating the operative time in minutes, with significance values included. References: [12,13,16] TAP: transversus abdominis plane

Operative time (minutes): Three studies reported on the operating time of abdominoplasties when a TAP block was used compared to when a TAP block was not used. The use of TAP block was associated with a shorter mean operating time by a difference of 30.86 min, but this was not statistically significant (95%CI -75.80 to 14.07 min, p = 0.18) [12,13,16]. Results are shown in Figure 6.

Discussion 

Postoperative pain management is an important element in perioperative patient care, with a reduction in discomfort directly leading to improved patient outcomes [20]. TAP blocks have been reported to mitigate the adverse effects associated with opiates in abdominoplasty procedures [3,11-16]. The authors present the first meta-analysis within the literature assessing the efficacy of TAP blocks in abdominoplasties and demonstrate a significantly lower time to first analgesic consumption in the TAP block group compared to no TAP block usage. In addition, an OR assessment of postoperative nausea and vomiting has shown a significantly lower incidence within the TAP cohort emphasising its efficacy. There was no significant difference in heterogeneity on assessment with the Cochrane Q test further giving consistency to this variable. Three studies reported on the operative time taken between the study and control groups with both showing an insignificant duration with the addition of a TAP block [12,13,16]. There were five studies commenting on the VAS, allowing patients to rate the intensity of pain [3,11,12,16,19]. Various scales were used, measured both during movement and at rest. Across all studies, a lower score was given by the patients who received the TAP block. No significant differences were evidenced for mean total narcotic usage as well as the number of pills consumed for both cohorts, although heterogeneity was quite high amongst these variables.

Abdallah et al. conducted a meta-analysis on the duration of analgesic effect following the posterior versus lateral approach to deliver a TAP block in various operations involving a lower abdominal incision. Across 12 RCTs comprising 641 patients, their findings revealed that prolonged analgesia is achieved from the posterior approach over the lateral approach [21]. This study does not directly observe the efficacy of TAP block on abdominoplasties, but it builds upon the evidence that administration of TAP block is more efficacious than no TAP block in abdominoplasty operations.

A systematic review by Opoku-Agyeman et al. provides complementary insights to studies that examined the administration of TAP blocks for cosmetic abdominoplasty surgeries. The study supports the efficacy of TAP block for overall pain control and its effect in reducing the consumption of postoperative narcotic drugs [22]. The study comments on the caveats to adapting TAP blocks more routinely into operations including the lack of training by anaesthesiologists, and a considerable variation in the technique, dosage, and timing of delivery. In addition, the study reported several techniques of direct administration, including a lateral and posterior approach [22]. This has included using different anaesthetic agents involving bupivacaine, liposomal bupivacaine, levobupivacaine, lidocaine and a mixture of both with epinephrine and dexamethasone. Some deliver this as a bolus whereas others as a continuous infusion following the bolus. TAP blocks can be administered both intraoperatively and postoperatively with no set guidelines. The administration technique is currently not standardised and ranges from ultrasound-guided to open muscle-splitting. A range of different approaches were reported within this review, with Abo-Zeid et al. utilising a mid-axillary infiltration whilst Alotaibi et al. used an ultrasound-guided technique [3,12]. It is noteworthy that this discrepancy was consistent throughout the review.

Five RCTs and three observational studies were included in this meta-analysis and the results have highlighted the benefits of utilising TAP blocks in abdominoplasties [3,11-16,19]. There are, however, inherent limitations associated with this review including the low number of RCTs, as well as the included observational studies drawing poor comparability scores on the Newcastle-Ottawa scale. Also, heterogeneity was high amongst a number of variables although this was circumvented by adopting a random effects model. The authors recommend more high-quality trials assessing the effects of TAP block administration in abdominoplasties with greater homogeneity in the anaesthetic agent used, method of administration, and technique to better evaluate its effectiveness.

## Conclusions

Pain impacts patient recovery and, therefore, postoperative care. This meta-analysis includes eight studies which demonstrate that the use of a TAP block for postoperative pain management following abdominoplasty is a significantly improved method of analgesia, with high heterogeneity between the variables. The results report a significantly lower time to the first analgesic consumption with a TAP block and a significantly lower incidence of postoperative nausea and vomiting. Across all studies, a lower score on the VAS was given by the patients who received the TAP block. The authors recommend more high-quality trials to further the current evidence base.
